# Lithobius (Monotarsobius) tetrasulcus sp. n., a new species of centipede from China (Lithobiomorpha, Lithobiidae)

**DOI:** 10.3897/BDJ.9.e73025

**Published:** 2021-09-28

**Authors:** Sujian Pei, Haipeng Liu, Kuijing Liang, Huiqin Ma, Yanmin Lu

**Affiliations:** 1 Institute of Myriapodology, School of Life Sciences, Hengshui University, Hengshui, China Institute of Myriapodology, School of Life Sciences, Hengshui University Hengshui China; 2 Hebei Key Laboratory of Wetland Ecology and Conservation, Hengshui, China Hebei Key Laboratory of Wetland Ecology and Conservation Hengshui China

**Keywords:** Hebei Province, Hengshui Lake National Nature Reserve, Chilopoda, Lithobiidae, *Monotarsobius* Verhoeff, 1905

## Abstract

**Background:**

The myriapod fauna of China is still poorly known and very little attention has been paid to the study of Lithobiomorpha, with only 100 species and subspecies known from the country. Altogether, 11 species of subgenusMonotarsobius have been recorded from China, but only two of them have been reported from Hebei Province. Herein, a new species recently discovered in the Hebei Province, China, is described and illustrated.

**New information:**

A new lithobiids species Lithobius (Monotarsobius) tetrasulcus sp. n. is described and illustrated from Hengshui Lake National Nature Reserve, Hebei Province, China. The new species is compared with Lithobius (Monotarsobius) crassipes Koch, 1862 from Taiwan, China. It can be easily distinguished from congeners by having a longitudinal groove on the dorsal side of the femur and tibia of the male legs 14 and 15, only having a posterior spine on the dorsal side of femur of legs 12–15, lacking robust spines lying dorsally on the external margin on the second article of the female gonopods and the third article of the female gonopods having a bidentate apical claw.

## Introduction

*Monotarsobius* was originally proposed as a subgenus of *Lithobius* Leach, 1814 in the family Lithobiidae by Verhoeff (Verhoeff 1905); it accommodates a group of 115 species and subspecies mostly known from a wide range of epigeic habitats, from low altitude sites to 4200 m, also caves of Asia, Europe and North Africa (Pocock 1895; Trotzina 1895; Attems 1901, Attems 1904; Dobroruka 1960, Dobroruka 1979; Zalesskaja 1978; Farzalieva and Zalesskaja 2002; Farzalieva 2006; Bonato et al. 2016; Zapparoli and Edgecombe 2011; Dányi and Tuf 2012). *Monotarsobius* is characterised by antennae with ca. 20 articles or thereabouts. Ocelli generally few, 1+1-1+11, absent in some cave species. Forcipular coxosternal teeth 2+2; porodonts setiform. Tergites without posterior triangular projections. Tarsal articulation of legs 1-13 very faint or indistinct. Secondary sexual modifications sometimes on male legs 14 and 15. Female gonopods with uni-, bi- or tridentate claw and usually 2+2 spurs (Zapparoli and Edgecombe 2011). Altogether, 11 species of *Monotarsobius* have been recorded from China, but only two of them have been reported from Hebei Province (Ma et al. 2014a, Ma et al. 2014b; Chao et al. 2018, Chao et al. 2020). Herein, a new species recently discovered in the Hebei Province, China, is described and illustrated.

## Materials and methods

Specimens were collected under leaf litter and/or stones and preserved in 75% ethanol. Illustrations and measurements were produced using a ZEISS SteREO Discovery.V20 microscope equipped with an Abbe drawing tube and an ocular micrometre and Axiocam 512 colour. The colour description is based on specimens fixed in 75% ethanol. The body length is measured from the anterior margin of the cephalic plate to the posterior end of the postpedal tergite. Type specimens and other material are deposited in the School of Life Sciences, Hengshui University, Hengshui, China (**HUSLS**). The terminology of the external anatomy follows Bonato et al. (2010). Measurements are shown in millimetres (mm). The following abbreviations are used in the text and the tables: a – anterior; C – coxa; F – femur; m – median; P – prefemur; p – posterior; S, SS – sternite, sternites; T, TT – tergite, tergites; Ti – tibia; Tr – trochanter.

## Taxon treatments

### Lithobius (Monotarsobius) tetrasulcus

Pei, Liu, Liang, Ma & Lu, 2021 sp. n.

E5E084DF-EF76-5E6F-927C-9E17FFF08C72

urn:lsid:zoobank.org:act:C42FBEA2-4099-46F2-856C-C5B5AA206509

#### Materials

- **Type status:**Holotype. **Occurrence:** catalogNumber: LETE01-1; recordedBy: Pei Sujian, et al.; individualCount: 1; sex: male; lifeStage: adult; **Taxon:** kingdom: Animal; phylum: Arthropoda; class: Chilopoda; order: Lithobiomorpha; family: Lithobiidae; genus: Lithobius; subgenus: Monotarsobius; specificEpithet: *tetrasulcus*; taxonomicStatus: species; **Location:** country: China; stateProvince: Hebei; county: Taocheng; locality: Beitian Village, Hebei Hengshui Lake National Nature Reserve; verbatimElevation: 24 m a.s.l.; locationRemarks: label transliteration: "Beitian Village, Hebei Hengshui Lake National Nature Reserve, Hebei Province, 21 April 2013, Sujian Pei, Huiqin Ma."; decimalLatitude: 37.647638; decimalLongitude: 115.665203; **Identification:** identifiedBy: Huiqin Ma; dateIdentified: 2020; **Event:** eventDate: 21/04/2013; **Record Level:** collectionCode: Myriapoda; basisOfRecord: Preserved Specimen
- **Type status:**Paratype. **Occurrence:** catalogNumber: LETE01-1; recordedBy: Pei Sujian, et al.; individualCount: 49; sex: 30 female and 19 male; lifeStage: adult; **Taxon:** kingdom: Animal; phylum: Arthropoda; class: Chilopoda; order: Lithobiomorpha; family: Lithobiidae; genus: Lithobius; subgenus: Monotarsobius; specificEpithet: *tetrasulcus*; taxonomicStatus: species; **Location:** country: China; stateProvince: Hebei; county: Taocheng; locality: Beitian Village, Hebei Hengshui Lake National Nature Reserve; verbatimElevation: 24 m a.s.l.; locationRemarks: label transliteration: "Beitian Village, Hebei Hengshui Lake National Nature Reserve, Hebei Province, 21 April 2013, Sujian Pei, Huiqin Ma."; decimalLatitude: 37.647638; decimalLongitude: 115.665203; **Identification:** identifiedBy: Huiqin Ma; dateIdentified: 2020; **Event:** eventDate: 21/04/2013; **Record Level:** collectionCode: Myriapoda; basisOfRecord: PreservedSpecimen
- **Type status:**Other material. **Occurrence:** catalogNumber: LETE01-2; recordedBy: Pei Sujian, et al.; individualCount: 11; sex: 6 males, 5 females; lifeStage: adult; **Taxon:** kingdom: Animal; phylum: Arthropoda; class: Chilopoda; order: Lithobiomorpha; family: Lithobiidae; genus: Lithobius; subgenus: Monotarsobius; specificEpithet: *tetrasulcus*; taxonomicStatus: species; **Location:** country: China; stateProvince: Hebei; county: Jizhou; locality: Weitun town, Jizhou County; verbatimElevation: 23 m a.s.l.; locationRemarks: label transliteration: "Weitun town, Jizhou County, Hengshui City, Hebei Province, 2 May 2019, Sujian Pei, Huiqin Ma."; decimalLatitude: 37.608275; decimalLongitude: 115.640952; **Identification:** identifiedBy: Huiqin Ma; dateIdentified: 2020; **Event:** eventDate: 02/05/2019; **Record Level:** collectionCode: Myriapoda; basisOfRecord: PreservedSpecimen
- **Type status:**Other material. **Occurrence:** catalogNumber: LETE01-3; recordedBy: Pei Sujian, et al.; individualCount: 8; sex: 2 males, 6 females; lifeStage: adult; **Taxon:** kingdom: Animal; phylum: Arthropoda; class: Chilopoda; order: Lithobiomorpha; family: Lithobiidae; genus: Lithobius; subgenus: Monotarsobius; specificEpithet: *tetrasulcus*; taxonomicStatus: species; **Location:** country: China; stateProvince: Hebei; county: Pingshan; locality: Xiushui Park, Pingshan County; verbatimElevation: 136 m a.s.l.; locationRemarks: label transliteration: "Xiushui Park, Pingshan County, Shijiazhuangi City, Hebei Province, 28 May 2017, Sujian Pei, Huiqin Ma."; decimalLatitude: 38.093677; decimalLongitude: 114.38752; **Identification:** identifiedBy: Huiqin Ma; dateIdentified: 2020; **Event:** eventDate: 28/05/2017; **Record Level:** collectionCode: Myriapoda; basisOfRecord: PreservedSpecimen

#### Description

Body: 11.9–17.0 mm long, cephalic plate 1.3–1.6 mm long, 1.4–1.8 mm wide.

Colour: The antennae are pale yellow-brown with pale greyish hue; tergites yellow-brown, cephalic plate, TT1, 14 and 15 darker brown; pleural region pale grey with pale yellow hue; sternites pale yellow-brown with pale greyish hue; distal part of forcipules darker brown, with basal and proximal parts of forcipules and forcipular coxosternite and SS14 and 15 yellow-brown; all legs pale grey with pale yellow hue; tibia of tarsus 1 darker yellow, tarsus 2 darkest yellow of all legs.

**Figure 1.**
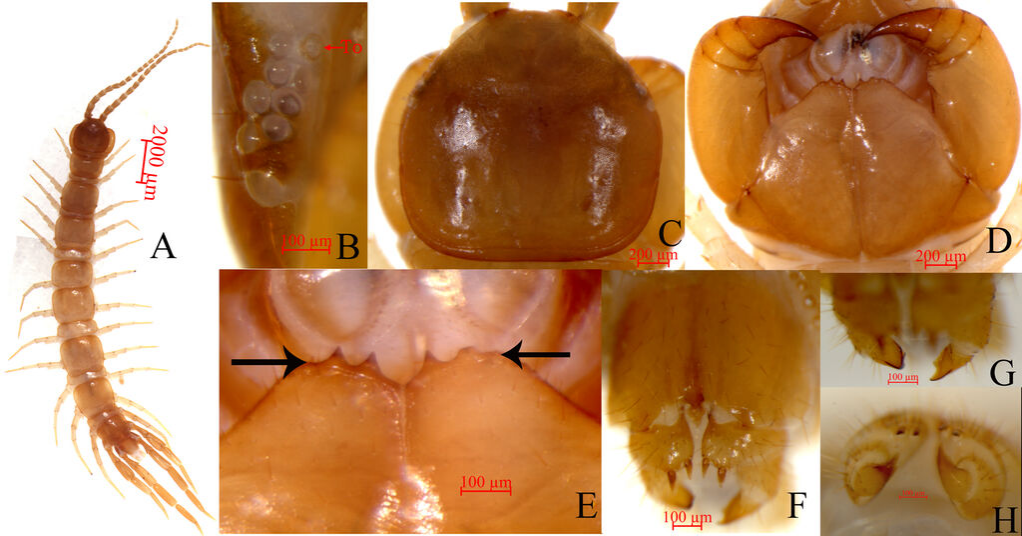
Lithobius (Monotarsobius) tetrasulcus sp. nov. **A** male holotype, habitus, dorsal view; **B** holotype, ocelli and Tomosvary’s organ (To), lateral view; **C** male holotype, cephalic plate, dorsal view; **D** male holotype, cephalic plate, ventral view; **E** male holotype, forcipular coxosternite, ventral view; **F** female paratype, posterior segments and gonopods, ventral view; **G** female paratype, apical claw of gonopods, ventral view; **H** female paratype, apical claw of gonopods, dorsal view.

Antennae bearing 19–21 articles, usually 20 articles (Fig. 1A). Length of first antennal article longer than width of the base, length of the remaining articles significantly larger than wide; from second article, each article is gradually shortened, the distal-most articles still significantly longer than wide, 2.3–2.9 times as long as wide; abundant setae on the antennal surface, less so on the basal articles, gradual increase in density of setae to approximately the fifth article, then more or less constant.

Cephalic plate smooth, convex, slightly wider than long; tiny setae emerging from pores scattered very sparsely over the whole surface; frontal marginal ridge with shallow anterior median furrow; short to long setae scattered along the marginal ridge of the cephalic plate; lateral marginal ridge discontinuous, posterior margin continuous, straight, slightly wider than lateral marginal ridge (Fig. 1C).

Ocelli. Eight to ten (commonly nine) oval to rounded ocelli on each side, from small to large, arranged in three irregular rows, the posterior ocellus largest. Ventral ocelli slightly smaller than the dorsal, domed, translucent and usually darkly pigmented (Fig. 1B).

Tömösváry’s organ (Fig. 1B, To) close to the ocelli, situated at the anterolateral margin of the cephalic plate, the surrounding sclerotised area is not obvious, moderately larger than the adjoining ocelli.

Forcipular coxosternite subtrapezoidal (Fig. 1D), anterior margin narrow, lateral margins slightly longer than medial margins; median diastema moderately shallow, wide V-shaped; anterior margin with 2+2 acute triangular teeth; porodonts slightly thicker, just posterolateral and separated from the lateral tooth, lying posteriolaterally to the lateral-most tooth (Fig. 1D and E); long scattered setae on the ventral side of coxosternite, longer setae near the dental margin.

Tergites smooth, without wrinkles, dorsum slightly convex; tiny setae emerging from pores scattered sparsely over the entire surface, near the margin with few long setae; T1 narrower postero-laterally than antero-laterally, generally inverted trapezoidal; TT1 and TT3 narrower than the cephalic plate, T3 wider than the T1. Lateral marginal ridges of all tergites continuous. Posterior margin of TT1, 3 and 5 continuous, posterior margin of TT8, 10, 12 and 14 discontinuous; posterior margin of T7 straight, posterior marginal ridges of TT3, 5, 8, 10, 12 and 14 with concavity gradually increasing. Posterior angles of tergites rounded, without triangular projections. Miniscule setae scattered sparsely over the surface, one thick and long setae on both anterior angles of each tergite.

Sternites. Posterior sides of sternites narrower than anterior, generally inverted trapezoidal, smooth; setae emerging from sparsely scattered pores on the surface and lateral margins, very few short setae sparsely scattered amongst them. Short and thick setae on the surface of the anterior part slightly increasing from S11 to S15.

Legs relatively robust, tarsi articulation ill-defined on legs 1–13, well-defined on legs 14 and 15. All legs with moderately long curved claws; legs 1–13 with anterior and posterior accessory spurs, anterior accessory spurs moderately long and slender, forming a moderately small angle with the claw, posterior accessory spurs slightly more robust, forming a comparatively large angle with the claw, legs 13 with anterior accessory spurs; anterior accessory spurs of legs 14 short and thick, posterior accessory spurs normal; legs 15 lacking accessory spurs. From short to long setae sparsely scattered over the surface of coxas, trochanters, prefemora, femora and tibiae of all legs, more setae on the tarsal surface; setae on the dorsal and ventral surfaces slightly longer than the anterior and posterior ones; legs 14 and 15 thicker than the anterior pairs in both female and male, legs 15 thicker and stronger in male than in female. Many glandular pores on the posterior surfaces of legs 14 and 15. Tarsus 2 is 3.5–4.4 times longer than wide, tarsus 2, 46.0%–69.1% length of tarsus 1 on legs 15 in female; tarsus 2, 3.9–4.9 times longer than wide, tarsus 2, 60%–70% length of tarsus 1 on legs 15 in male. Leg plectrotaxy as in Table 1.

Coxal pores 3–6 in a row, 4-5(6)-5-4, 3(4)-5-5(4)-3(4), 4-4(5)-4-4, commonly 4-5-5-4 in female, 3-4(5)-4-3(4), commonly 3-4-4-3 in male; slightly oval or round, coxal pore field set in a relatively shallow groove, the coxal pore-field fringe with slight prominence, with moderately long setae sparsely scattered over the surface.

Female. S15 anterior margin broader than posterior, generally inverted trapezoidal, pos­tero-medially straight. Moderately long setae sparsely scattered on S15 surface. Surface of the lateral sternal margin of genital segment well chitinised, posterior mar­gin of genital sternite deeply concave between condyles of gonopods, except for a small, median rhombic-shaped bulge. Relatively long setae very sparsely scattered over ventral surface of the genital segment, slightly more setae on posterior part, especially on the posterior edge. Gonopods: first article fairly broad, bearing 16 or 17 moderately long setae, arranged in three irregular rows; with 2+2 small coniform spurs, inner spur slightly smaller than the outer (Fig. 1F); second article with 10–12 long setae in the ventral side, arranged in three irregular rows; third article with three or four long setae on the ventral side, with a bidentate apical claw (Fig. 1G and H).

**Figure 2.**
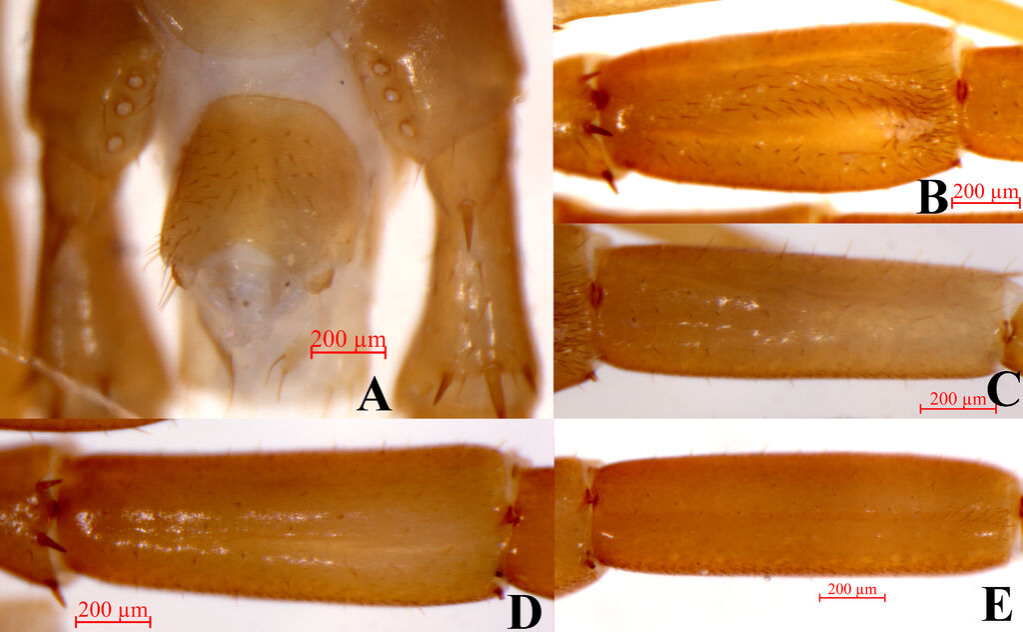
Lithobius (Monotarsobius) tetrasulcus sp. nov., holotype, male **A** posterior segments and gonopods, ventral view; **B** femur of legs 14, dorsal view; **C** tibia of legs 14, dorsal view; **D** femur of legs 15, dorsal view; **E** tibia of legs 15, dorsal view.

Male. S15 posterior margin narrower than anterior, postero-medially straight, sparsely covered with long setae; sternite of genital segment evidently smaller than the female, usually sclerotised; posterior margin deeply concave between the gonopods, without medial bulge. Short to long setae evenly scattered on the ventral surface of the genital segment. Gonopods short, appearing as small finger-like bulges, with 2–4 long setae, apically slightly sclerotised (Fig. 2A). With a wide central longitudinal groove on the dorsal side of femur of the male legs 14, the setae on both sides of the groove slightly increasing in number gradually posteriorly and the terminal setae obviously dense (Fig. 2B); a narrow central longitudinal groove extending to the middle of the dorsal of tibia of the male legs 14 (Fig. 2C); a narrow central longitudinal groove extending to the terminal of the dorsal femur and tibia of the male legs 15; the setae on both sides of the groove slightly increased in number (Fig. 2D and E).

#### Diagnosis

Antennae composed of 19–21 articles, ocelli 8–10, usually nine on each side, arranged in three irregular rows, the posterior ocellus comparatively large, Tömösváry’s organ larger than the adjacent ocelli; commonly 2+2 coxosternal teeth, porodonts lying posterolateral to the lateral-most tooth; coxal pore formula 3–6, arranged in one row; legs 14 and 15 are obviously modified in male, with a central longitudinal continuous groove on the dorsal of femur and tibia. Female gonopods with 2+2 moderately small coniform spurs, apical claw of the third article bidentate; male gonopods short and small.

#### Etymology

To emphasise the obviously central longitudinal continuous groove on the dorsal of femur and tibia of the male legs 14 and 15.

#### Taxon discussion

The new species is morphologically close to Lithobius (Monotarsobius) crassipes Koch, 1862 (Koch 1862) from Taiwan, China, with which it shares 8–10 ocelli on each side arranged in three irregular rows, the posterior ocellus largest, the Tömösváry’s organ larger than the adjoining ocelli, 2+2 prosternal teeth, a coxal pore formula of 3–6 and female gonopods with 2+2 coniform spurs. However, they can be distinguished easily by the following characters. The new species has a longitudinal groove on the dorsal femur and tibia of the male legs 14 and 15 vs. no longitudinal groove on the dorsal of femur and tibia in L. (M.) crassipes; with posterior spine on the dorsal of femur of legs 12–15, other than legs 10–14 in L. (M.) crassipes; lacking robust spines lying dorsally on the external margin on the second article of the female gonopods in contrast to the presence of three robust spines in L. (M.) crassipes; lacking robust spines lying dorsally on the external margin on the third article of the female gonopods in contrast to the presence of one robust spine in L. (M.) crassipes.

## Supplementary Material

XML Treatment for Lithobius (Monotarsobius) tetrasulcus

## Figures and Tables

**Table 1.** Leg plectrotaxy of Lithobius (Monotarsobius) tetrasulcus sp. nov.

**legs**	**ventral**					**dorsal**				
	**C**	**Tr**	**P**	**F**	**Ti**	**C**	**Tr**	**P**	**F**	**Ti**
1			p	amp	am			ap	ap	a
2			mp	amp	am			ap	ap	ap
3			mp	amp	am			ap	ap	ap
4			mp	amp	am			a(m)p	ap	ap
5-11			mp	amp	am			amp	ap	ap
12			mp	amp	am	(a)		amp	p	p
13		(m)	amp	amp	am	a		amp	p	p
14		m	amp	amp	a(m)	a		amp	p	
15		m	amp	am	a	a		amp	p	

Letters in brackets indicate variable spines.
